# Supplementary material for: Hyperactivation of MAPK Induces Tamoxifen Resistance in SPRED2-Deficient ERα-Positive Breast Cancer
Source: Cancers (Basel). 2022 Feb 14;14(4):954. doi: 10.3390/cancers14040954 (PMC8870665; doi:10.3390/cancers14040954)
Supplement: Supplementary file 1 [file cancers-14-00954-s001.zip › cancers-1544688-supplementary.pdf]

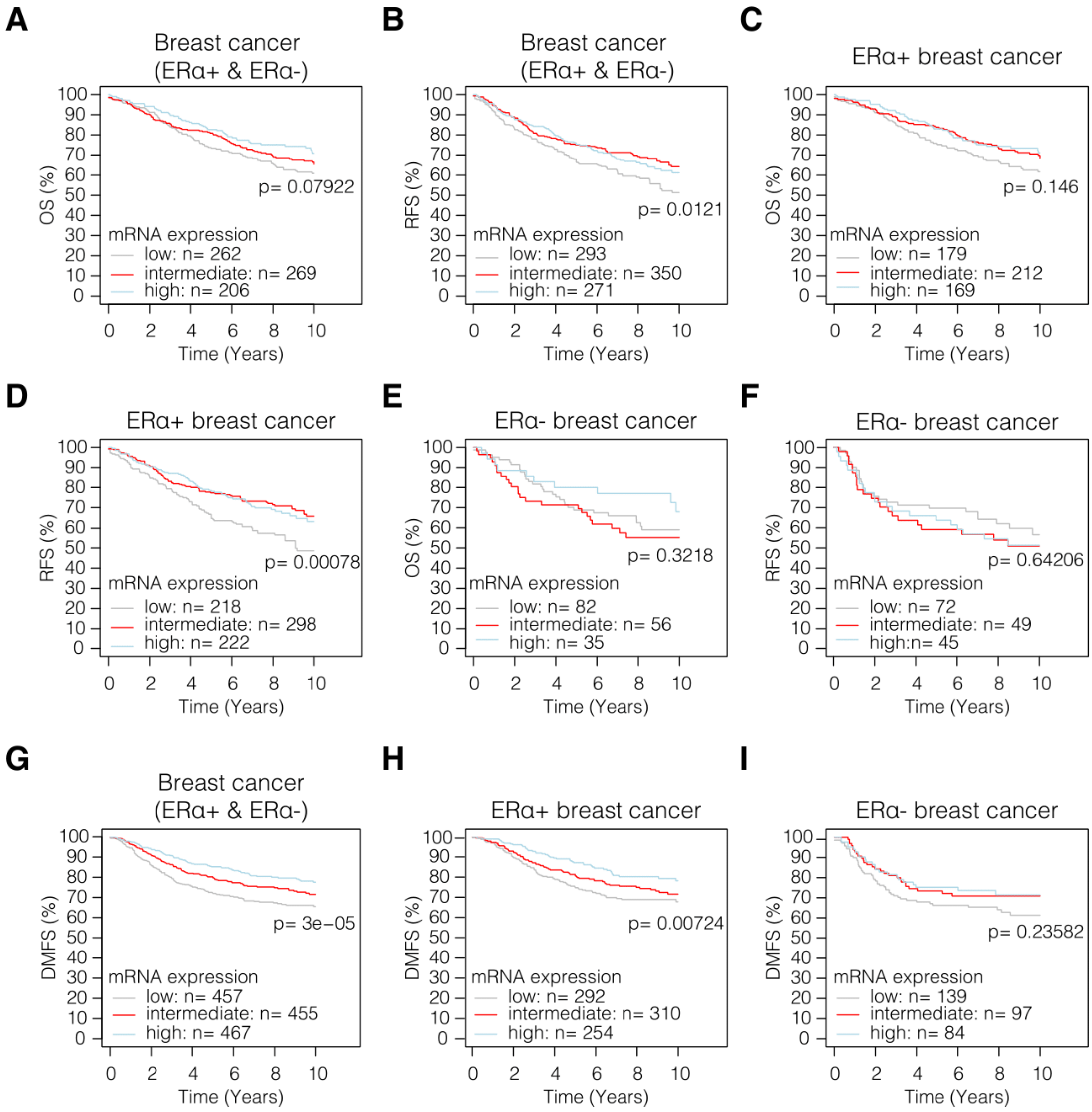

Figure S1. Low *SPRED2* gene expression correlates with poor survival of patients with breast tumors. (**A-F**) Kaplan-Meier plots derived from the GOBO database for OS and RFS of all (**A,B**), ERα+ (**C,D**) and ERα– (**E,F**) breast cancer patients classified as having tumors expressing high levels (blue line), intermediate levels (red line) and low levels (grey line) of *SPRED2* mRNA. (**G-E**) Kaplan-Meier plots derived from the GOBO database for distant metastasis free survival (DMFS) of all (**G**) ERα+ (**H**) and ERα– (**I**) breast cancer patients classified as above.

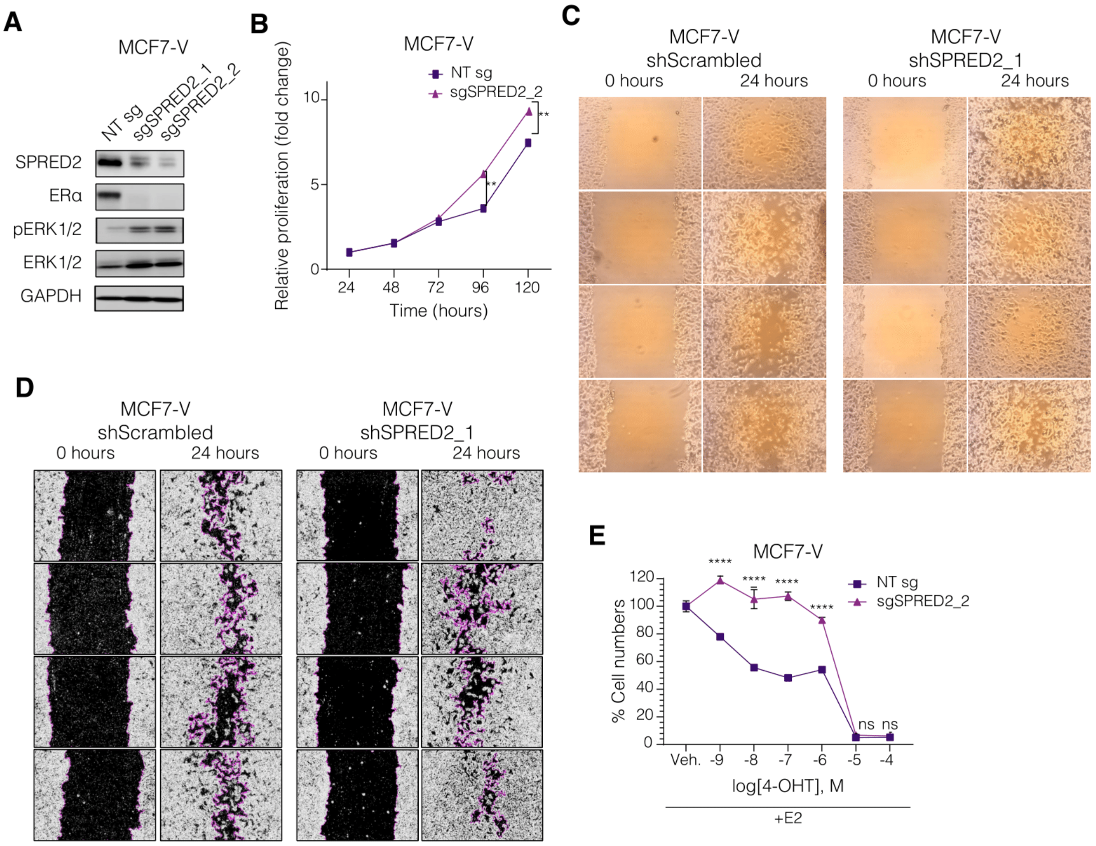

Figure S2. *SPRED2* deficiency enhances cell proliferation, migration, and induces resistance to 4-OHT in ERα+ BC cells. (**A**) Representative immunoblots with specific antibodies showing the efficiency of reducing SPRED2 levels in MCF7-V cells with the CRISPR/Cas9 system, as well as the protein levels of ERα and MAPK-related proteins. Note that SPRED2 protein is not completely eliminated indicating only partial sgSPRED2-mediated knockouts. Uncropped images of immunoblots with molecular weight standards are in Figure S5. (**B**) Relative proliferation of MCF7-V clones expressing either a non-targeting control (NT sg) or SPRED2-targeted (sgSPRED2) sgRNA, measured by crystal violet assay in monolayer cultures. The number of cells for each of the them are standardized to the corresponding values at 24 h set to 1. (**C**,**D**) Raw (**C**) and ImageJ-edited images (**D**) of the four replicates each of the scratch wound healing assays with MCF7-V cells. (**E**) Dose-response curves of MCF7-V cells, with sgRNA-mediated depletion of SPRED2, upon treatment with increasing doses of 4-OHT. Asterisks indicate significant differences compared to the NT sg control (ns for $p > 0.05$, * $p \leq 0.05$, ** $p \leq 0.01$, *** $p \leq 0.001$ and **** $p \leq 0.0001$). Statistical significance was determined with a two-way ANOVA.

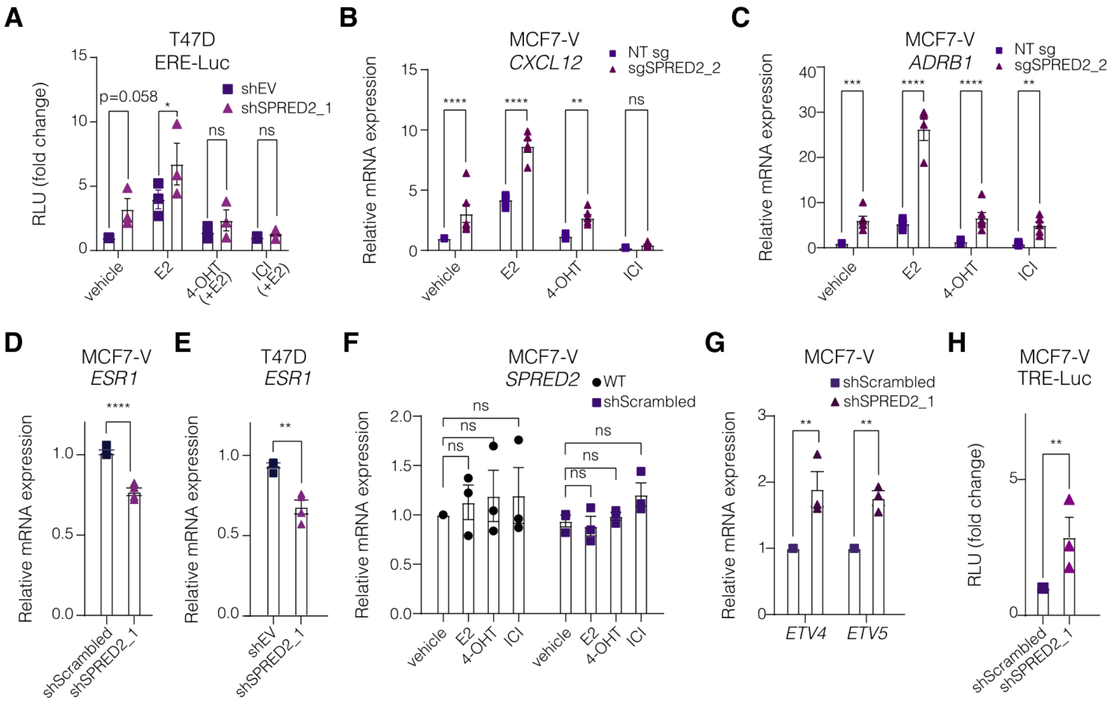

Figure S3. Knockdown of *SPRED2* stimulates ERα transcriptional activity by increasing the activity of the MAPKs ERK1/2. (**A**) Luciferase reporter assay with transiently transfected T47D cells. The activity of endogenous ERα was monitored with the ERE-Luc reporter plasmid. RLUs are relative to the activities of the internal transfection standard Renilla luciferase. Graphs are based on n=3 independent experiments. (**B,C**) Expression of ERα target genes in MCF7-V sgRNA clones; mRNA levels were analyzed by RT-qPCR following 6 h of treatments as indicated; n=3 independent experiments. (**D,E**) Expression levels of *ESR1* (ERα) mRNA determined by RT-qPCR with the indicated MCF7-V (**D**) and T47D (**E**) samples. (**F**) Expression levels of *SPRED2* mRNA in the untransfected parent (WT) MCF7-V cells and in shScrambled MCF7-V cells, measured with RT-qPCR. (**G**) Expression levels of the ERK1/2 target genes *ETV4* and *ETV5* in MCF7-V cells as indicated, measured with RT-qPCR. (**H**) Luciferase reporter assay with transiently transfected MCF7-V cells expressing the indicated shRNAs. The activity of AP-1 was monitored with the TRE-Luc reporter plasmid and expressed as RLU relative to the activities of the internal transfection standard Renilla luciferase. Graphs are based on n=3 independent experiments. All error bars represent the standard errors of the means (mean ± SEM). For luciferase assays, asterisks indicate significant differences compared to control cells transduced with empty vector (shEV) (T47D) or viruses for expression of negative control shRNA (shScrambled) (MCF7-V). The data of each bar graph were standardized to the values of the vehicle-treated non-targeted control samples set to 1. For RT-qPCR graphs, asterisks indicate significant differences compared to the NT sg cells (ns for $p > 0.05$, * $p \leq 0.05$, ** $p \leq 0.01$, *** $p \leq 0.001$ and **** $p \leq 0.0001$). Statistical significance was determined with a two-way ANOVA
(**F**) and a two-tailed unpaired t-test for all other panels.

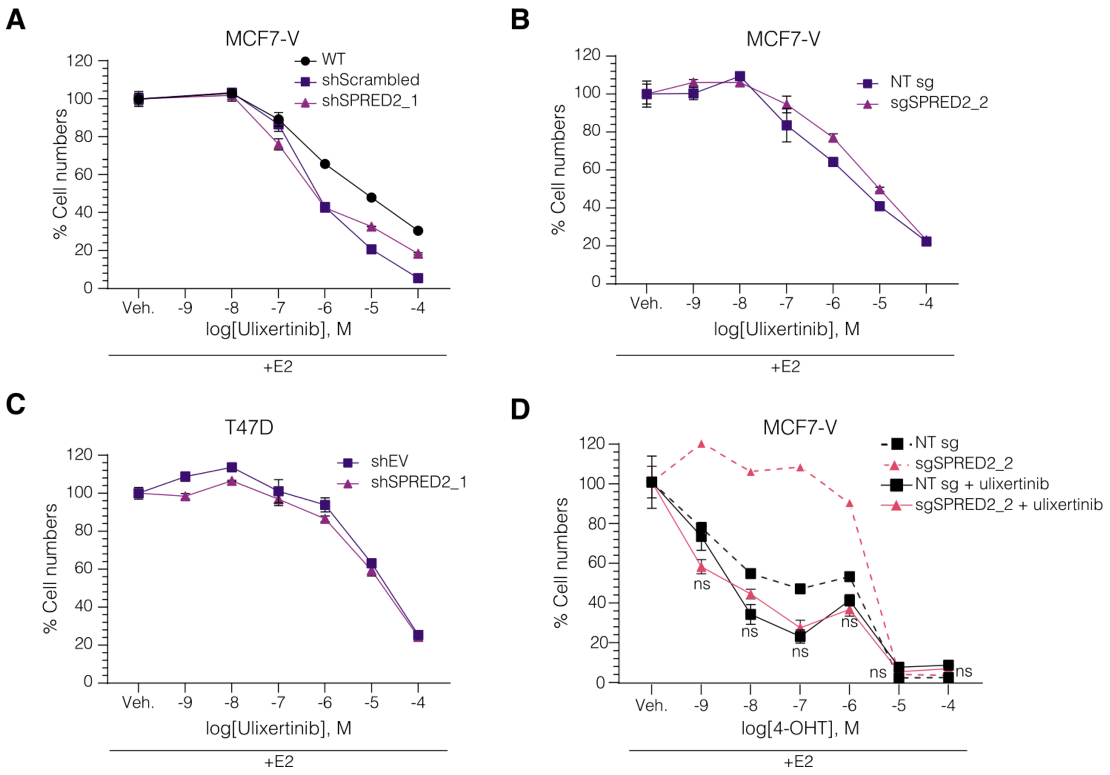

Figure S4. Ulixertinib resensitizes SPRED2-deficient BC cells to 4-OHT. (**A-C**) Dose- response curves with increasing doses of ulixertinib in MCF7-V (**A,B**) and T47D (**C**) cells. IC10 to IC20 doses of ulixertinib were chosen for combination treatments, i.e. 100 nM for MCF7-V and 300 nM for T47D cells. WT, parent MCF7-V cells. (**D**) Dose-response curves with increasing doses of 4-OHT in combination with 100 nM ulixertinib for MCF7-V. The dashed curves indicate the response to 4-OHT without ulixertinib, copied from Supplementary Figure S2E for comparison. All error bars represent the standard errors of the means (mean ± SEM). Asterisks indicate significant differences compared to the NT sg cells (ns $p > 0.05$, * $p \leq 0.05$, ** $p \leq 0.01$, *** $p \leq 0.001$ and **** $p \leq 0.0001$). Statistical significance was determined with a two-way ANOVA.

Figure S5. Uncropped images of immunoblots with molecular weight standards.

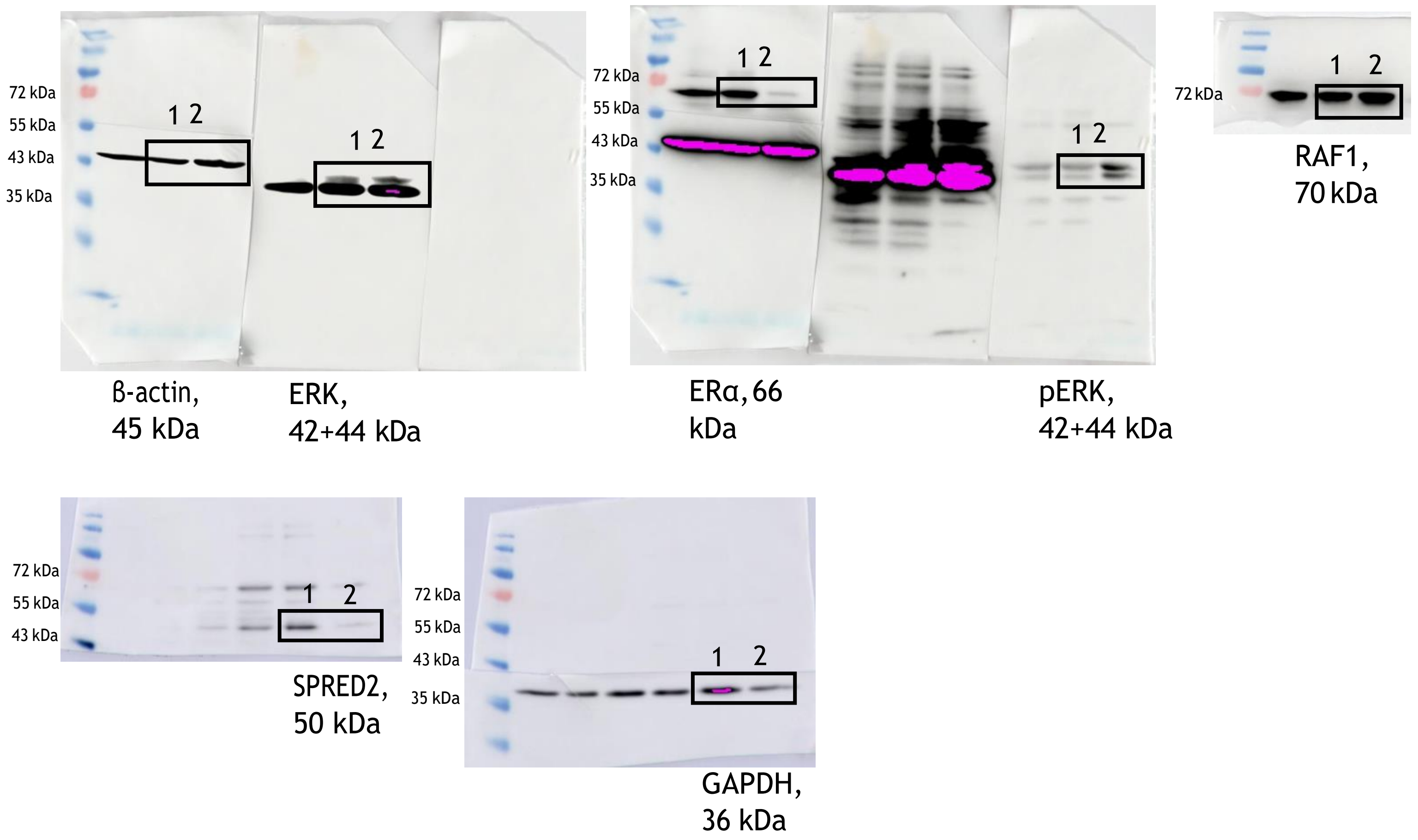

Figure S3A:
MCF7-V

1: shScr
2: shSPRED2

Figure S3B: T47D

1: shEV
2: shSPRED2

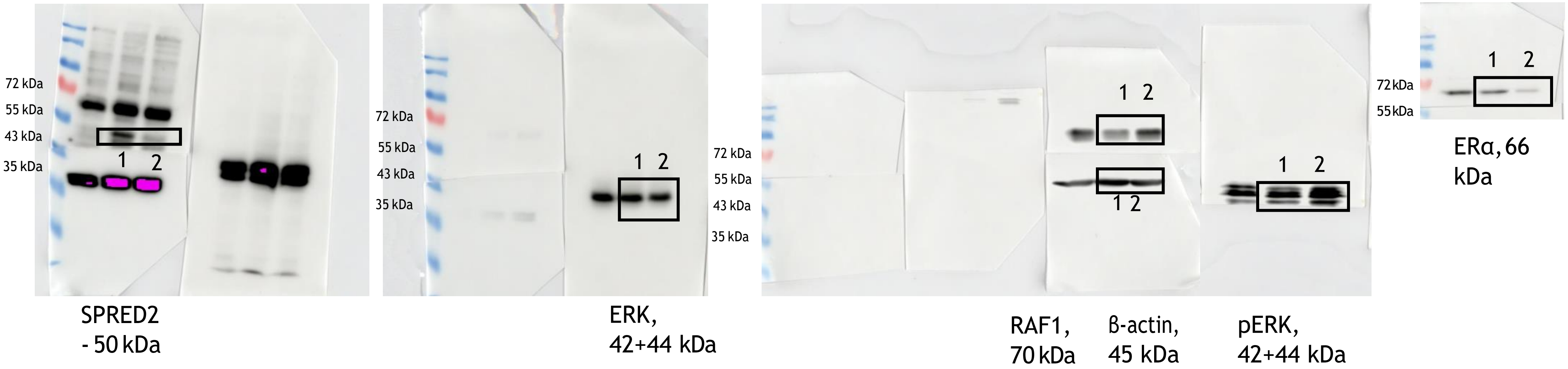

Figure S2A: MCF7-V

1: NT sg

2: sgSPRED2 (1)

3: sgSPRED2 (2)

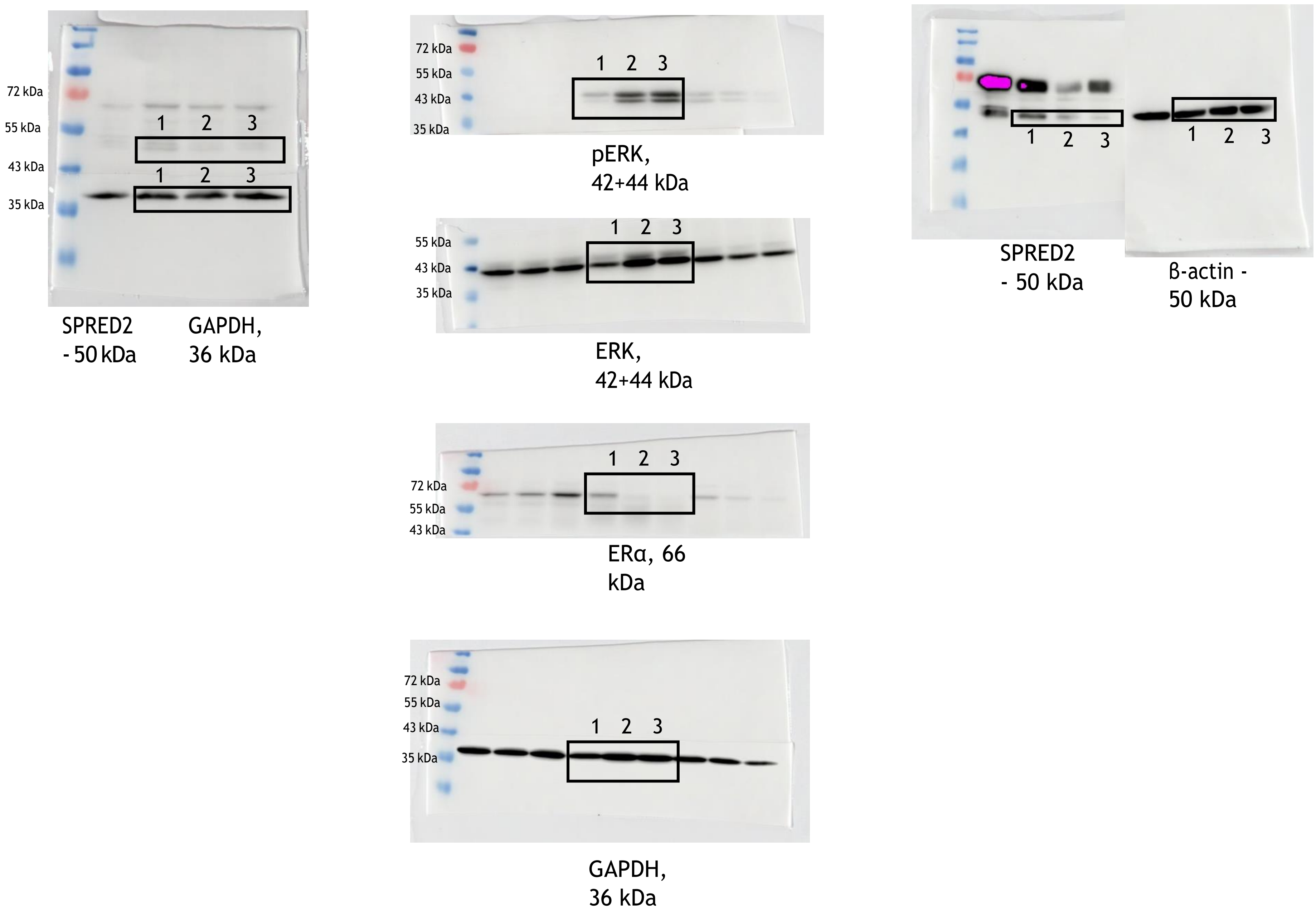

Figure S3J,K: HEK293T

1: HEK WT

2: shScr

3: shSPRED2

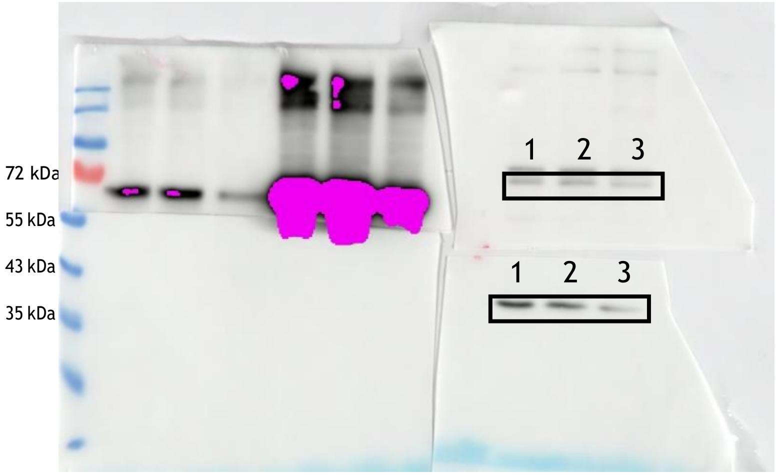

SPRED2 - 50 kDa

GAPDH, 36 kDa

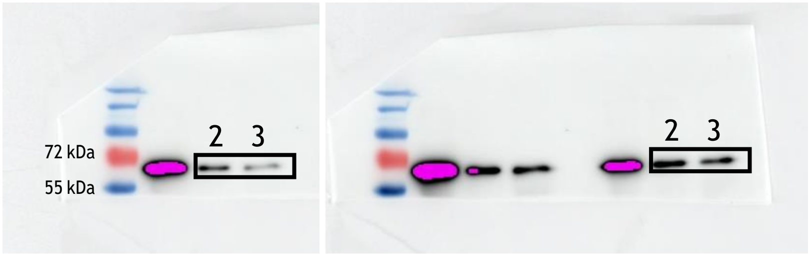

ERα-HEG0, 66 kDa

ERα-S118A, 66 kDa

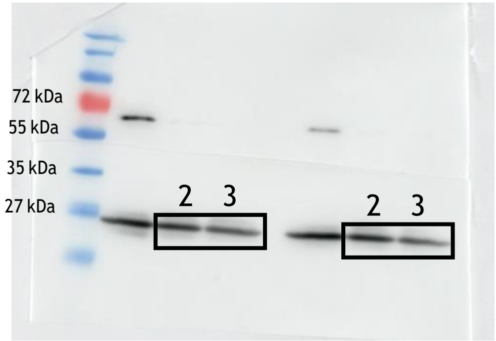

GFP-HEG0, 27 kDa

GFP-S118A, 27 kDa

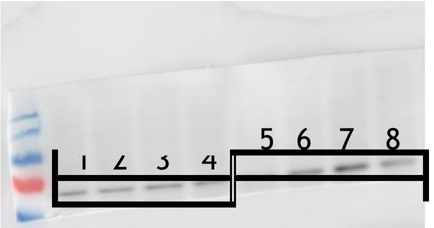

ERα, 66 kDa

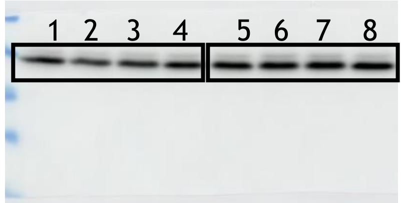

ERK, 42+44kDa

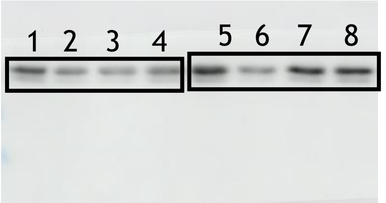

pERK, 42+44kDa

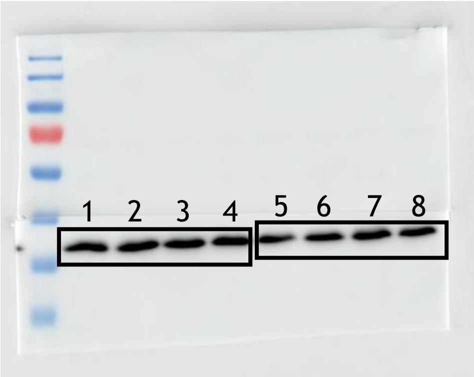

GAPDH, 36 kDa

Figure S4C: MCF7-V

1: shScr-veh

2: shScr-Uli

3: shScr- 4-OHT

4: shScr- Uli+4-OHT

5: shSPRED2-veh

6: shSPRED2- Uli

7: shSPRED2- 4-OHT

8: shSPRED2- Uli+4-OHT
